# Hierarchically porous materials and green chemistry—an interview with Ming-Yuan He

**DOI:** 10.1093/nsr/nwaa131

**Published:** 2020-06-15

**Authors:** Li-Hua Chen

**Affiliations:** Professor at Wuhan University of Technology

## Abstract

Many examples of hierarchies are present in nature, such as water transport in leaf vein systems, the respiratory system, the blood circulatory system, etc. Hierarchically structuring a material over different length scales by mimicking natural systems can provide an opportunity to render the material suitable for a variety of functions. Tremendous research over the past decade has focused on the synthesis and applications of hierarchically structured porous materials. This rapidly evolving field has attracted great interest from both academia and industry. China is at the forefront of this field, and a scientific leader of this research is Professor Ming-Yuan He of East China Normal University. Professor He was elected to the Chinese Academy of Sciences in 1995, and he received the Prize for Scientific and Technological Progress from the Ho Leung Ho Li Foundation in 2001. He also won the National Catalysis Achievement Award of China in 2012 and the National Zeolite Lifetime Achievement Award of China in 2019. Professor He's research interests focus on new catalytic materials and oil-refining catalysts and processes. He is a pioneer in the area of green chemistry in China and actively promotes the development of green chemistry and chemical engineering.

NSR recently interviewed Professor He about the current achievements and future prospects of hierarchically structured porous materials. This interview is dedicated to Professor He on the occasion of his 80th birthday, in recognition of his distinguished contributions to many aspects in the field of catalytic science and technology.

**NSR:** Hierarchies are present in many places in our environment, such as in biological systems from simple unicellular organisms to the more complex human body. Could you please discuss the definition of porous hierarchy? What are hierarchically structured porous materials?

**He:** In a broad sense, porous materials are those materials possessing pore structure with a bi- or multimodal pore size distribution, irrespective of whether or how these two (or more) different pore systems are interconnected. Bi- or multimodal porous systems can be specified as ‘hierarchical’ only if the overall pore system shows a well-ranked porous structure that increases mass transport. Thus, it should be noted that the term ‘hierarchically structured porous material’ is specifically used to draw a clear distinction between this type of material and the broader term ‘multi-porous material’.

In March 2017, Professor Bao-Lian Su, Professor Zai-Ku Xie, and I co-chaired the 590th Xiangshan Science Conference in Beijing. At this conference, hierarchically structured porous materials were defined as materials with a porous structure consisting of interconnected pores on different length scales including micropores (<2 nm), mesopores (2–50 nm) and macropores (>50 nm). In hierarchically structured porous materials, the micropores provide size and shape selectivity for guest molecules, the mesopores increase micropore accessibility, and the macropores provide unimpeded transport paths. This pore hierarchy is particularly important for the diffusion of large molecules or in viscous systems. In short, the criteria of hierarchically structured porous materials are *multiple levels*, *interconnectivity* and *regularity*.

**Figure:**
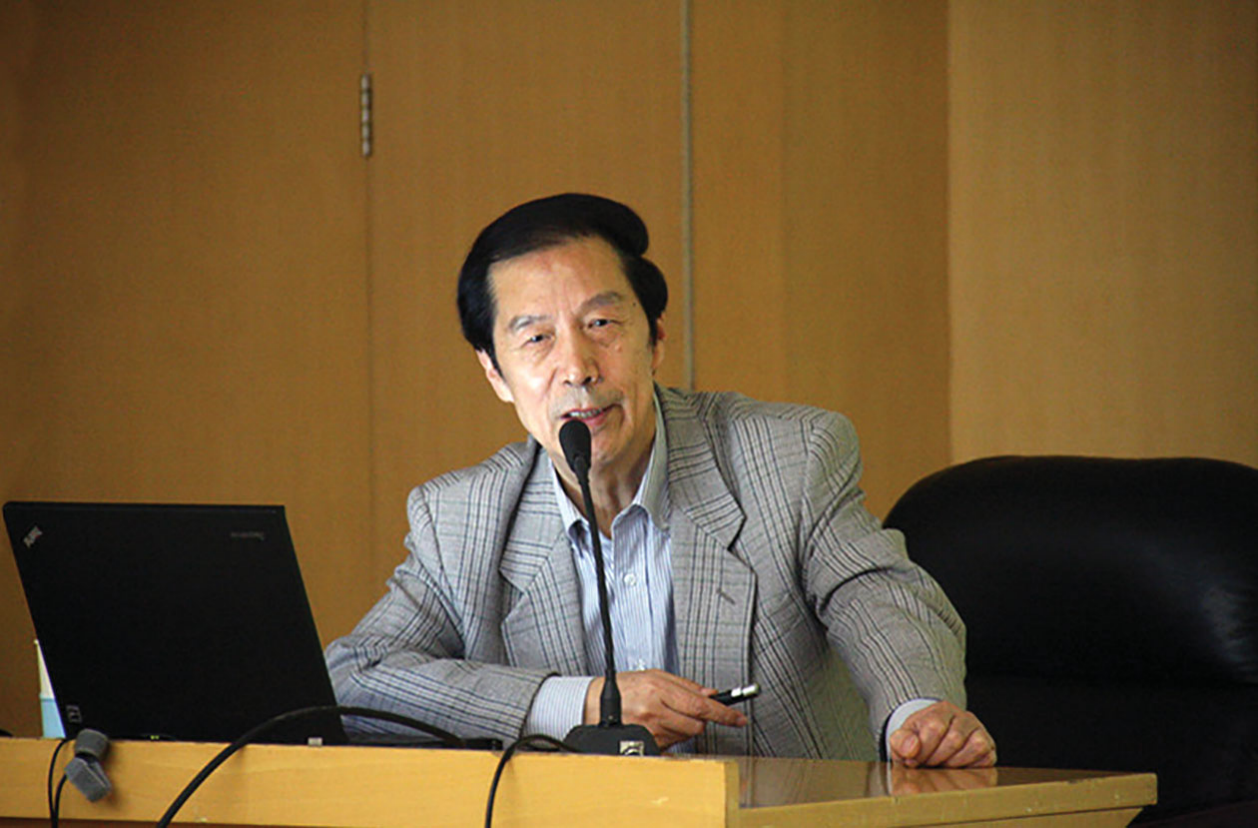
Professor Ming-Yuan He is a green chemistry pioneer at East China Normal University (*Courtesy of Professor Ming-Yuan He*).

**NSR:** Multiple fields ranging from biotechnology, biomedicine, catalysis, energy, optics, and separation to biomolecule immobilization and bio-organisms have a fervent interest in hierarchically structured porous materials. What do you think are some of the most important questions today in research on hierarchically structured porous materials?

**He:** I think that the first topic to address is the need to develop new synthesis strategies for the future design of

The criteria of hierarchically structured porous materials are multiple levels, interconnectivity, and regularity.

—Ming-Yuan He

tailored hierarchical architectures and the precise control over the connectivity between different pore dimensions. In general, hierarchically structured porous materials offer large surface area and effective mass transport of reactants and products to/from active sites located inside their frameworks. However, many hierarchically structured porous materials contain disordered pore networks. This disorder affects the nature of catalytically active sites, such as acid strength/density and surface hydrophobicity, compared with that of purely microporous analogues. The second important topic is the development of new synthetic strategies to realize large-scale production of hierarchically structured porous materials. Most of the recently synthesized hierarchically structured porous materials are in powder form, which means that they cannot be directly used in practical applications. Methodological limitations currently prevent the large-scale synthesis of hierarchically structured porous materials, which seriously restricts their further use and development.

**NSR:** As a green carbon science pioneer, could you please summarize green carbon science?

**He:** The concept of green carbon science was first proposed by Professor Yu-Han Sun, Professor Bu-Xing Han and myself, and presented at the 15th National Catalysis Conference in 2010 as a plenary lecture and summarized in a scientific paper. Carbon is the key element of life, society and industry. Carbon energy resources are extremely important for the foreseeable future of human society and are especially important for the energy strategy of our country. People should be always aware that the appropriate existence of carbon dioxide (CO_2_) and the greenhouse effect is necessary for life on earth. The essential factors regarding CO_2_ and the greenhouse effect are therefore *balance* and *recycling*. Carbon, hydrogen and oxygen are the three main chemical elements involved in carbon energy resources. Carbon energy utilization and carbon neutral balance are achieved based on the chemical principles of oxidation and reduction. Thus, as a natural rule, *hierarchy* is a characteristic of carbon energy resources. According to the ternary chemical composition of hydrogen, carbon and oxygen, when carbon energy is taken as a criterion, different carbon energy resources can be ranked in the following sequence: natural gas, petroleum, coal, biomass and CO_2_. Green sustainable chemistry is now proved to play a creative role in addressing and solving the world's energy trilemma: sustainable development – energy – environment. If we replace the term ‘energy’ by ‘carbon energy’ and ‘environment’ by ‘carbon dioxide’, then a novel concept ‘green carbon science’ is emerging, which more precisely focuses on the trilemma of sustainable development – carbon energy – carbon dioxide.

Sustainable development is a great challenge for our society. Improving the efficiency of carbon resource utilization and carbon recycling can contribute substantially to meeting this challenge. Highly efficient transformations and use of fossil reserves, biomass and CO_2_ with minimized energy consumption and CO_2_ emission are all essential. Accordingly, green carbon science is the study and optimization of the transformation of carbon-containing compounds and the relevant processes involved in the entire carbon cycle including carbon resource processing, carbon energy utilization, CO_2_ fixation and carbon recycling to use carbon resources efficiently and minimize net CO_2_ emission.

Sustainable development is an important issue for humans and a great challenge. Chemically feasible and effective solutions to achieve a sustainable society are the efficient use of limited fossil resources and the development of processes to convert biomass into fuels and value-added chemicals on a large scale.

**NSR:** What is your favorite topic in green carbon science?

**He:** It is commonly recognized that the ultimate energy source for human beings is solar energy. I am highly interested by the extent to which green carbon science can play a role in solar energy utilization. One important topic in green carbon science is biomass conversion. The efficient use of carbon resources, decreasing carbon emissions, and carbon recycling have become strategic research focal points and are great challenges for society. The processing and use of fossil resources and biomass conversion have been studied extensively. At present, most fuels and energy used are derived from non-renewable fossil resources. Sustainable development is an important goal for humans but is a great challenge. The development of biomass conversion is an effective solution to this problem. Biomass is an abundant renewable carbon source.

**NSR:** What are the challenges in green carbon science?

**He:** Many current routes to transform biomass are technically feasible but economically prohibitive, and need to be further improved to achieve efficient and low-energy-consumption processes on a large scale. In addition, the unique structure of biomass offers great opportunities to design and produce new chemicals. Attention should be paid to designing new synthetic strategies to form products with desired properties that maintain the structures of the feedstocks as much as possible. In this way, we can obtain the products required with low energy consumption. Many new valuable products, which are currently not produced from fossil resources, are expected to be synthesized in the future from biomass.

**NSR:** Are hierarchically structured porous materials important to promote the development of green carbon science?

**He:** Yes, hierarchically structured porous materials are important in the progress of green carbon science. Currently, most fuels and energy used are derived from fossil resources, and fossil

The essential factors regarding CO_2_ and the greenhouse effect are therefore balance and recycling.

—Ming-Yuan He

energy will continue to be the dominant energy source in the foreseeable future. The highly efficient utilization of the limited petroleum resources is extremely important because we have no alternative sources and technologies to provide the tremendous amount of liquid transportation fuels we need at present. Designing hierarchically porous catalyst materials to minimize the generation of coke and CO_2_ under industrial conditions is critical.

Biomass provides a huge amount of renewable carbon. Efficient transformation of biomass into high-quality fuels and chemicals is a long-standing task. Typically, biomass conversion involves the decomposition of bulky biopolymers into small basic molecules and then often the further processing of these small molecules to form value-added end-products. The accelerated diffusion performance and improved accessibility to active sites achieved using hierarchically structured porous materials help to facilitate biomass conversion. Thus, hierarchically porous materials play an important role in many biomass conversions routes. Hierarchically structuring both the porosity and architecture of a material over different length scales provides the opportunity to render the material suitable for a variety of functions that are desirable for green carbon science.

**NSR:** Despite substantial progress in the preparation of hierarchically porous materials, it could be argued that hierarchical porous structure cannot easily be designed and synthesized for a specific application. Do you have any suggestions or perspectives on the design theory for hierarchically porous materials?

**He:** At present, we still follow the process of trial, testing, modification and retesting to reach optimized, but not the best, target materials. This situation is because of the lack of principles, rules and theories on the design of advanced materials. ‘Material properties by design’ is an attractive concept for future development. Is it possible to establish material design principles to achieve predictive, optimized functions? I think that nature could yield important inspiration by providing concrete examples to help identify rules to follow and emulate. Many classes of organisms have hierarchically porous networks with extremely high efficiency and minimum energy consumption, such as plant stems, leaf veins, and vascular and respiratory systems. These living hierarchically porous networks connected within a finite volume can minimize the transport resistance

New discoveries and concepts quite often reside in the experimental facts that do not conform to expectations.

—Ming-Yuan He

to all the pores and ensure fluent transfer throughout the entire hierarchical network as a precondition for organisms.

Using the generalized Murray's law, Prof. Bao-Lian Su's group recently fabricated the first family of bioinspired materials emulating natural vascular structure. The pore sizes decreased across multiple scales and finally terminated in a size-invariant unit resembling the hierarchical structure of plant stems, leaf veins, and vascular and respiratory systems. These biomimics possessed hierarchical branching and strongly promoted mass exchange and transfer in liquid–solid, gas–solid and electrochemical reactions. This is the first example of quantitative materials design and synthesis of diameter ratios to connect multiscale pores from macro to micro levels. This work is a pioneering demonstration of the synthesis of simultaneously optimized multi-length-scale materials following design rules that evolved in natural hierarchical systems and that enable the functionality of material networks to be predictably controlled.

It is highly desirable to use this approach to construct other hierarchical networks of interconnected pores within materials. I am pleased to see that many industrial efforts have been devoted to this field; for example, the Shanghai Institute of Petrochemical Technology (Sinopec), led by Professor Zai-Ku Xie and Professor Wei-Min Yang, has put considerable effort into this field from the aspect of industrial applications. The concept of hierarchical materials has also been applied to metal-organic frameworks and fibers. I hope that this special topic of *National Science Review* focusing on hierarchically structured porous materials will attract much new research attention to boost progress in this field from laboratory to industry.

**NSR:** What suggestions would you make to young researchers entering the field of hierarchically porous materials?

**He:** Hierarchically porous materials have been extensively studied for many years and numerous achievements have been made. Young researchers should think carefully about what they can do before entering this field. I advise that they choose emerging or unsolved challenges as their focus based on their background and interests. They will need to combine other concepts such as confinement, shape selectivity and molecular recognition with hierarchically structured porous materials. New ideas come from the interface between scientific knowledge and unanswered questions. New discoveries and concepts quite often reside in the experimental facts that do not conform to expectations. Young researchers should always keep their curiosity and investigate what they are really interested in.

